# Modulating the Expression of Disease Genes with RNA-Based Therapy

**DOI:** 10.1371/journal.pgen.0030109

**Published:** 2007-06-29

**Authors:** Matthew Wood, Haifang Yin, Graham McClorey

**Affiliations:** University College London, United Kingdom

## Abstract

Conventional gene therapy has focused largely on gene replacement in target cells. However, progress from basic research to the clinic has been slow for reasons relating principally to the challenges of heterologous DNA delivery and regulation in vivo. Alternative approaches targeting RNA have the potential to circumvent some of these difficulties, particularly as the active therapeutic molecules are usually short oligonucleotides and the target gene transcript is under endogenous regulation. RNA-based strategies offer a series of novel therapeutic applications, including altered processing of the target pre-mRNA transcript, reprogramming of genetic defects through mRNA repair, and the targeted silencing of allele- or isoform-specific gene transcripts. This review examines the potential of RNA therapeutics, focusing on antisense oligonucleotide modification of pre-mRNA splicing, methods for pre-mRNA *trans*-splicing, and the isoform- and allele-specific applications of RNA interference.

## Introduction

RNA targeting is emerging as a powerful alternative to conventional gene replacement therapies for the treatment of genetic disorders. Although an emerging field, RNA modification has the potential to circumvent some of the shortcomings of standard gene therapy methods, including: (i) low efficiency of gene transfer; (ii) limitations on transgene size, specifically an inability to deliver genomic size loci; (iii) insertional mutagenesis and integration-associated events; and (iv) immune responses and toxicity due to vectors. Moreover, some disease situations could be more amenable to correction by RNA targeting, such as autosomal dominant diseases, where introduction of a functional gene does not address expression of the dominant mutant transcript. Similarly, in disorders of RNA processing, such as aberrant splicing, it may be preferable to repair the endogenous splicing pattern, which could also correct multiple alternative isoforms. More importantly, RNA targeting has unique potential for therapeutic modification of native mRNA transcripts within a normal regulatory environment. The potential of such approaches ranges from elimination of the mRNA in question to modification of the mature mRNA product by the removal or addition of natural elements or exons and to repair of the mRNA transcript by the addition of foreign mRNA elements to create a chimeric gene product. Many of the effector molecules underpinning these novel methods have their origins in natural biochemical pathways that have been discovered in recent years.

## Antisense Oligonucleotide-Based Manipulation of Pre-mRNA Splicing

Traditionally, antisense oligonucleotides (AOs) have been employed to down-regulate gene transcription, through either RNase-H mediated degradation or steric blockage of gene promoter elements. More recently however, the potential of using AOs to alter pre-mRNA processing is being realised. Through utilisation of AO chemistries that do not induce transcript degradation, targeted blockage of motifs involved in splicing allows the manipulation of this process. Given that in the Human Gene Mutation Database (http://www.hgmd.cf.ac.uk/ac/index.php) ∼10% of annotated mutations impinge on splice sites [1], there is the potential for this approach to be applied to diseases caused by aberrant splicing, or where alteration of normal splicing would abrogate the disease-causing mutation. This could include: (i) blockage of cryptic splice sites, (ii) exon removal or inclusion to alter isoform expression, and (iii) removal of exons to either eliminate a nonsense mutation or restore the reading frame around a genomic deletion.

### 

#### 

##### Blockage of cryptic splicing.

**Figure 1:**
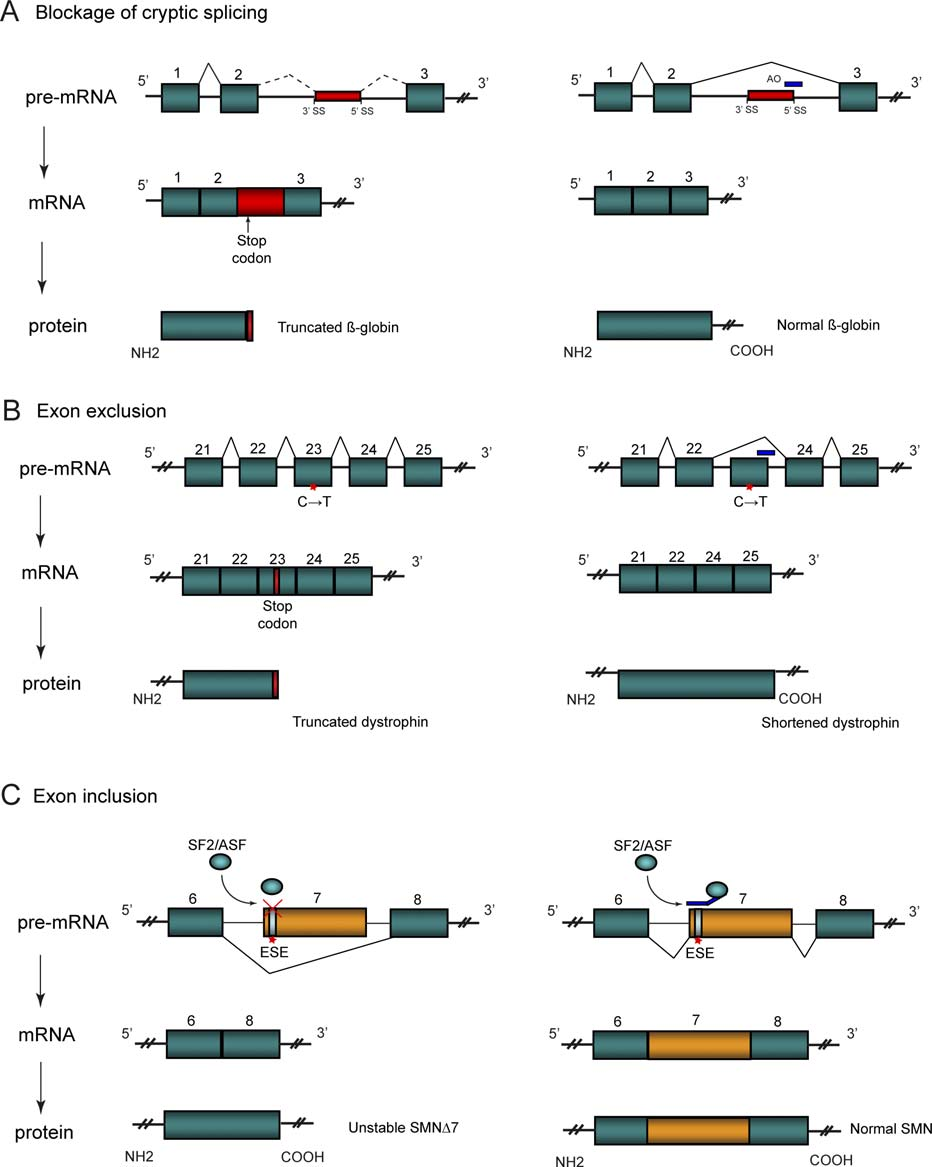
AO-Based Manipulation of Pre-mRNA Splicing (A) Blockage of cryptic splicing as a therapy for β-thalassemia. Mutations within intron 2 of the β-globin gene induce usage of cryptic splice sites that incorporate intronic sequence into the mature mRNA. Disruption of the reading frame introduces a stop codon that results in truncated β-globin protein. Blockage of the cryptic 5′ splice site with AO (blue bar) restores normal splicing pattern and functional β-globin protein is produced. (B) Restoration of dystrophin production in the mouse model of DMD by exon exclusion. A C→T mutation in exon 23 of the mouse dystrophin gene introduces a stop codon that produces a truncated nonfunctional protein. Blockage of the 5′ splice site of exon 23 disrupts its recognition by splicing machinery, resulting in removal of the in-frame exon from the dystrophin transcript. This facilitates translation of near full-length, semi-functional dystrophin protein. (C) Exon inclusion to increase production of SMN protein as a therapy for SMA. A silent mutation in the *SMN2* gene disrupts an ESE site in exon 7, preventing binding of the SF2/ASF splicing factor and affecting exon recognition such that the majority of *SMN2* transcripts lack exon 7, producing a poorly functional SMN protein. Targeting with a bifunctional AO (blue bar) containing a functional ESE sequence recruits the SF2/ASF factor, promoting exon recognition and incorporation into the mature transcript, resulting in translation of normal SMN protein.

The concept of using AOs to alter pre-mRNA splicing was first demonstrated in β-thalassemia, which is caused by mutations in the human β-globin gene [2]. The most prevalent disease-causing mutations disrupt splicing of introns 1 and 2 of the β-globin pre-mRNA transcript through activation of cryptic splice sites, preventing production and translation of the correct mRNA [3]. Blockage of these cryptic splice sites with AOs in erythroid cells abrogates their use, restoring normal globin expression [4] (Figure 1A). Similarly, in Hutchinson-Gilford progeria syndrome, a point mutation in exon 11 of the lamin A/C *(LMNA)* gene causes a silent substitution (GGC→GGT) that results in activation of a cryptic donor splice site [5]. Splicing between this cryptic splice site and the acceptor splice site gives rise to a truncated *LMNA* mRNA containing a 50-aa internal deletion in the globular tail domain of lamin A [5]. Targeting of the Hutchinson-Gilford progeria syndrome mutation region with a 25-mer morpholino AO sterically blocks the activated cryptic splice site, resulting in >90% reduction in the mutant lamin at both RNA and protein level in Hutchinson-Gilford progeria syndrome–derived fibroblasts in vitro [6].

##### Exon exclusion.

The demonstration that AOs could block the utilisation of cryptic splice sites led to the idea that modification of constitutive splicing could remodel disease-associated pre-mRNA transcripts. In Duchenne muscular dystrophy (DMD), nonsense or deletion mutations in the dystrophin gene disrupt translation of a functional dystrophin protein [7]. A less severe allelic form of the disease, Becker muscular dystrophy, has in-frame deleted transcripts that allow the synthesis of a shorter yet partially functional dystrophin protein [8]. Potentially, blockage of consensus splice sites or exon splicing enhancers (ESEs) by modified AOs to exclude exons, could be used to partially correct the disease by converting DMD-causing dystrophin transcripts to the milder Becker-like transcripts. In the *Dmd* mouse model, a nonsense mutation in exon 23 induces a premature termination in translation. Removal of this in-frame exon by targeting of AOs to the 5′ donor splice site of intron 23 facilitates the synthesis of a near full-length Becker muscular dystrophy–like protein that localises correctly to the sarcolemmal membrane [9] (Figure 1B). Systemic intravenous administration of 2′-*O*-methyl phosphorothioate AOs with the copolymer F127 demonstrates low levels of dystrophin positive fibres in multiple muscle groups with increased expression observed following repeat injections [10]. This was further improved using morpholino AO chemistry, with both increased dystrophin expression [11,12] and functional improvement, as evident by increased muscle maximum isometric tetanic force and a reduction in creatine kinase levels [11]. This AO-based approach has also been applied to human in vitro models of DMD, where targeted removal of single or multiple exons has been used to restore the reading frame around genomic deletions in DMD patient–derived myotube cultures, such that a near full-length dystrophin protein was induced [13,14]. The success of these experiments, and the demonstration of dystrophin transcript correction in human DMD muscle explants [15], suggests great promise for this approach as a therapy for DMD, with Phase I clinical trials recently underway.

In the microtubule-associated protein tau *(MAPT)* gene, alternative splicing in exons 2, 3, and 10 of the *MAPT* pre-mRNA results in expression of six isoforms in the brain [16]. Exon 10 splicing is regulated by multiple *cis*-acting elements such that exclusion or inclusion gives rise to tau isoforms with three (tau3R) or four (tau4R) microtubule binding repeats, respectively [17]. In adult brain, these isoforms are expressed in equal amounts, but in patients with frontotemporal dementia with parkinsonism linked to Chromosome 17, mutations in the *MAPT* gene affects exon 10 retention such that a 2–6-fold excess of tau4R over tau3R occurs [18,19]. This in turn is thought to affect microtubule properties, as tau4R and tau3R have different qualitative and quantitative effects on microtubule dynamics. Conventional *MAPT* cDNA replacement to address this isoform imbalance is complicated by the toxicity of tau overexpression [20]. To demonstrate the potential of exon inclusion as a treatment for frontotemporal dementia with parkinsonism linked to chromosome 17, AOs were directed against the splice junctions of exon 10 of *MAPT* pre-mRNA to efficiently induce its exclusion in an in vitro system [21].

##### Exon inclusion.

Just as targeted blockage of consensus splice sites and ESEs promotes exon exclusion, the blockage of exonic or intronic splicing silencers, or the introduction of splicing enhancer sequences, can enhance exon inclusion [1]. This offers the potential to enhance expression of alternatively spliced “weak” exons to induce the most functionally preferable isoform. In spinal muscular atrophy (SMA), mutations in the survival motor neuron *(SMN1)* gene are responsible for a degenerative disease that presents as childhood muscle weakness and, in the more serious forms, can cause fatal respiratory failure [22]. The severity of the disease is modified by the production of SMN protein encoded by the paralogous gene, *SMN2* [23]. Although *SMN2* is nearly identical to *SMN1,* a silent C→T mutation in exon 7 abrogates an ESE site [24], weakening recognition of the upstream 3′ splice site [25] and resulting in the majority of *SMN2* transcripts lacking exon 7. As this SMNΔ7 isoform is unstable, and at best, only partially functional [26], the level of full-length SMN protein is an important modifier of patient disease severity. A number of AO-based strategies have been developed to promote exon 7 inclusion in the *SMN2* transcript. The concept of competition between the 5′ splice site of exon 6 and the 3′ splice sites of exons 7 and 8 led to the notion that blockage of the exon 8 acceptor splice site would promote splicing between exon 6 and 7 [25]. Transfection of 2′-*O*-methyl phosphorothioate AOs [25] and modified U7 small nuclear RNA [27] to target the intron 7/exon 8 junction results in increased exon 7 inclusion in *SMN2* transcripts and a subsequent increase in full-length SMN protein. Manipulation of exon 7 inclusion was also demonstrated through AO-mediated blockage of intronic splicing silencers within intron 6 [28] and immediately upstream of the 5′ splice site of exon 7, with increased SMN protein levels demonstrated in SMA patient derived cells [29]. Oligonucleotides were also designed to artificially introduce splicing elements so as to promote inclusion of a target exon. These AOs consist of two parts; an antisense sequence that targets to the specific exon, and a noncomplementary tail with RNA sequences that correspond to ESE sequences that are recognised by splicing proteins. Transfection of these AOs into SMA patient fibroblasts increases the level of endogenous *SMN2* containing exon 7 from ∼60% to ∼85%, the level observed in normal individuals [30] (Figure 1C). Bifunctional AOs have also been designed with a target-specific antisense moiety coupled to a peptide domain designed to mimic the splicing activation domains of serine/arginine–rich proteins. The ability of these oligonucleotides to stimulate exon 7 inclusion was demonstrated in cell-free splicing assays [31] and also in SMA type I fibroblasts, with an increase in SMN protein levels observed [32].

As well as restoring protein function through inclusion of an exon essential for function, it is possible to bias natural alternative splicing for the desired outcome. Apoptosis is a highly regulated process, with the relative expression levels of pro- and anti-apoptotic genes thought to be particularly important. Deregulation of this process is one of the hallmarks of the development and maintenance of cancer, and is often due to splicing defects in these regulatory genes. The *bcl-x* gene (also known as *BCL2L1*) has two splicing variants, the pro-apoptotic *bcl-xS* and the anti-apoptotic *bcl-xL,* which is overexpressed in various cancers [33]. Blocking of the alternative 5′ splice site in intron 2 of *bcl-x* with AOs shifts splicing from the *bcl-xL* to the *bcl-xS* isoform, with a subsequent increase in apoptotic markers in cancer cell lines [34]. Efficiency of this process is improved further when this splice site is targeted with a bifunctional AO consisting of an antisense moiety coupled to a heterogeneous nuclear ribonucleoprotein A1 exon silencing motif [35].

Modified AOs are a powerful tool for manipulating alternative splicing and addressing disease-causing splicing defects; however, there are limitations to the use of this approach. Exon exclusion can only be applied when the induced in-frame deletion lies in a region that is noncritical for function. In the majority of genes, a deletion in the mRNA transcript will disrupt protein function, rendering this approach unsuitable. Re-administration of AOs would also be a necessity, as these compounds have a limited biological half-life and only transiently target the gene transcript and not the gene. To address this limitation, recombinant AAV vectors were constructed to stably express modified small nuclear RNAs containing antisense sequences against the *Dmd* mouse mutation [36]. Interarterial perfusion in the lower limb of the *Dmd* mouse resulted in more than 80% expression of dystrophin-positive fibres and coincided with functional improvement [36]. However, host immune responses to viral proteins would limit the repeat administration, which may be necessary, as muscles undergo cycles of degeneration and regeneration. Similarly, viral vectors have been designed to express bifunctional oligonucleotides containing potent exonic enhancers to promote exon 7 inclusion in the *SMN2* gene for treatment of SMA [37]. The potential off-target effects of application of AOs in vivo have not been well studied and remain an important issue for consideration. Although statistically the chances of binding of a typical 25-base oligomer to a nonspecific gene target are low, this potentially increases when targeting common splicing motifs or when the antisense binding region size is decreased, as is the case for many bifunctional oligonucleotides. There is also the consideration of toxicity due to the AO chemistry employed or its associated carrier, which would need to be carefully monitored in animal models before application in human clinical trials.

## RNA *Trans*-Splicing

Rather than modify pre-mRNA splicing, the emergence of RNA *trans*-splicing has allowed methods to be developed for repairing genetic defects in the mature mRNA transcript. *Trans*-splicing is a natural process, although rare in mammals, which involves splicing between two separately transcribed mRNAs such that a composite transcript is produced. Manipulation of this process offers the potential for induction of isoform switching or the correction of dominant mutations by conversion to a wild type gene product. The most common methodologies in current use are spliceosome mediated RNA *trans*-splicing (SMaRT; Intronn proprietary technology, http://www.intronn.com) and ribozyme mediated *trans*-splicing.

### 

#### SMaRT.

**Figure 2:**
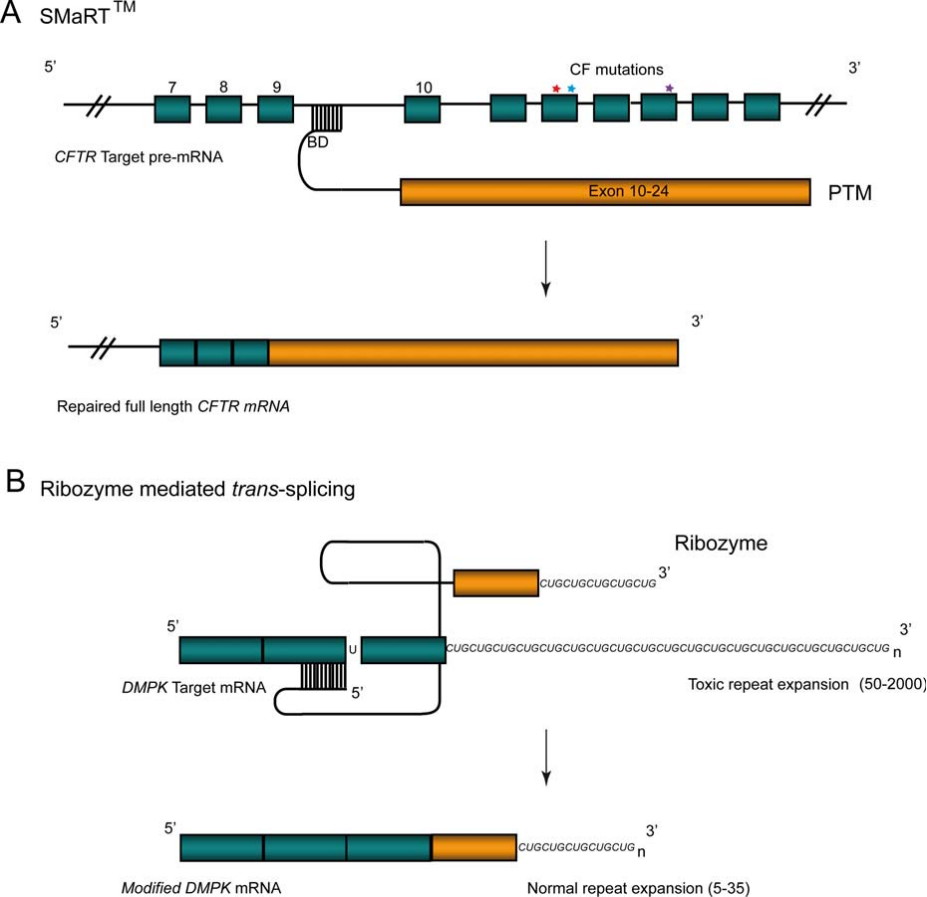
RNA *Trans*-Splicing (A) Correction of CF mutations in the *CFTR* gene using SMaRT. A PTM containing a binding domain (BD), splicing domain (black line), and a coding domain (orange) incorporating exons 10–24 of wild-type *CFTR* mRNA, binds to intron 9 of *CFTR* pre-mRNA (green) containing disease-causing mutations (stars). SMaRT removes the mutant pre-mRNA such that reprogrammed transcript containing wild-type mRNA allows synthesis of a functional protein. (B) Ribozyme-mediated *trans*-splicing for application to trinucleotide repeat expansions. Large (50–2,000) CUG repeat expansion in the 3′ untranslated region of the *DMPK* gene cause myotonic dystrophy. Ribozymes containing a reduced number of CUG repeats are targeted to the mutant *DMPK* transcript (green) via complementary binding mediated by a guide sequence (black bars). Binding of the ribozyme facilitates cleavage of the *DMPK* mRNA and *trans-*splicing of the coding region (orange) and smaller CTG repeat expansion to produce a non-toxic *DMPK* mRNA transcript.

In this approach, an engineered pre-mRNA *trans*-splicing molecule (PTM) binds specifically to target pre-mRNA in the nucleus such that it triggers *trans*-splicing in a process mediated by the spliceosome [38]. The major components of the PTM are a binding domain, a splicing domain, and a coding domain. The binding domain confers target specificity, whereas the splicing domain contains motifs necessary for the *trans*-splicing reaction to occur. The coding domain carries the portion of the wild-type cDNA, usually one or more exons, that are necessary to repair the targeted mutation. This repair is typically achieved by exon replacement and subsequent removal of the defective portion of the target pre-mRNA so that a functional gene product can be transcribed (Figure 2A). Functional correction using spliceosome-mediated *trans*-splicing has been reported in several preclinical disease models, including cystic fibrosis (CF) [39], haemophilia A [40], and X-linked immunodeficiency [41].

One of the hurdles in gene therapy for CF is the necessity of regulating cystic fibrosis transmembrane conductance regulator *(CFTR)* gene expression in the appropriate cell types in the airway. Studies of overexpression of *CFTR* have suggested that it may actually be deleterious to airway cells, so a strategy of ubiquitous expression of recombinant *CFTR* may not be ideal [42]. However, as the products of *trans*-splicing are dependent on cellular machinery for their expression, localisation to the correct cell subset would be expected. The potential of SMaRT *trans*-splicing has been shown in vitro and in vivo in a ΔF508 model of CF, the most prevalent disease-causing mutation [39]. Adenoviral vectors carrying a PTM engineered to bind to intron 9 of the *CFTR* pre-mRNA and *trans*-splice exons 10–24 of wild-type *CFTR* were transfected into either human CF airway epithelial cultures or in bronchial xenografts, resulting in functional correction of chloride transport of 16%–22% [39]. Adenoviral vectors are unlikely to be clinically applicable in this context, however, due to their poor ability at transducing the airway epithelia, so this approach was tested using a recombinant adeno-associated virus (AAV) delivery system [43]. Functional activity reached approximately 14% of that observed in non-CF epithelia, similar to the adenoviral vector studies. With low immunogenicity and the ability to infect multiple tissue types, recombinant AAV makes an attractive vector for gene therapy. Additionally, the potential limitation in AAV vector packaging capacity is less of an issue with SMaRT *trans*-splicing, as only a portion of the *CFTR* cDNA needs to be delivered.

The majority of *trans*-splicing studies to date have focused on restoration of function through replacement of the portion of the mRNA transcript containing the disease-causing mutation. However, *trans*-splicing also has potential application in treating disorders linked to aberrant splicing. To investigate regulation of alternative splicing of human *MAPT* exon 10, a PTM carrying exons 10–13 of the *MAPT* coding region was designed to bind to *MAPT* intron 9 [44]. Following cotransfection into human neuroblastoma cells with a tau mini-gene containing exons 9–11, the increase in the ratio of exon 10 inclusion to exclusion indicated approximately 34% efficiency of *trans*-splicing [44]. These results demonstrate that SMaRT can be used to manipulate alternative splicing and could have therapeutic application for those disorders that are a consequence of aberrant splicing.

SMaRT has several advantages over conventional gene therapy. As the gene is repaired rather than introduced, the spatial and temporal expression of the gene should be controlled by endogenous regulation such that protein expression resembles that for normal individuals. As repair will only occur where the target transcript is expressed, adverse effects would not be anticipated in cells that were nonspecifically targeted during delivery. *Trans*-splicing can also address autosomal dominant disorders. As the level of repaired transcripts increases, the level of mutant transcript would be expected to decrease, which gene replacement does not address. Another advantage is that because only a fragment of the gene needs to be replaced, the PTM constructs are easily accommodated in current vector systems. One of the major disadvantages is that a single PTM, in most cases, would not be able to address all mutations in an affected population. There is also the potential for nonspecific *trans*-splicing [45]; however, improvements in PTM design, especially with regard to the binding domain, have increased their specificity [46]. While cotransfection of mini-gene targets and PTMs have obtained reasonable levels of *trans*-splicing in vitro, for endogenous pre-mRNA or stably expressed pre-mRNA in vivo, splicing efficiency is lower [40]. Further optimisation of PTM design will be necessary for high *trans*-splicing efficiency and, as with all RNA therapeutic approaches, development of efficient methods of delivery to the cells where repair is required is essential before this approach could be considered clinically applicable.

#### Ribozyme-mediated *trans*-splicing.

Derived from the naturally occurring Group I self-splicing introns, *trans*-splicing ribozymes consist of a guide sequence complementary to the target sequence, the ribozyme domain, and a 3′ terminal exon that is to be *trans*-spliced. Following binding, the ribozyme catalyses *trans*-splicing between the 3′ exons of the ribozyme and the 5′ target mRNA (Figure 2B). Ribozyme-mediated repair was first demonstrated for β-globin mRNA in erythroid precursors from individuals with sickle cell disease [47]. In myotonic dystrophy, the most common neuromuscular disease in adults, increased levels of trinucleotide repeat expansion in the 3′ untranslated region of the dystrophia myotonica-protein kinase (*DMPK*) gene, are responsible for the clinical condition [48]. To demonstrate the feasibility of addressing expansion repeat mutations using *trans*-splicing ribozymes, a specifically designed ribozyme was used to reduce the number of repeats from 12 to five, at the 3′ end of *DMPK* transcripts in mammalian cells [49]. This methodology could be applied to the repair of other repeat expansion mutations; however, the relatively low efficiency means that it is not yet suitable for clinical application. Ribozyme-mediated *trans*-splicing has also been applied in other disease models in vitro, including p53 and p16 mutations in human ovarian cancer [50] and pancreatic cancer cell lines [51], respectively. In these studies, ribozymes were developed to be capable of repairing any mutations in the coding region of the gene. Although efficiencies were low, as many of these mutations exert a dominant-negative effect, each *trans*-spliced mRNA molecule would reduce expression of the mutant protein while simultaneously increasing wild-type protein expression.

As well as correcting disease-causing mutations, *trans*-splicing ribozymes have the potential to create chimeric gene transcripts by splicing foreign cDNA to a targeted mRNA. This was recently demonstrated in targeting cancer cells that express carcinoembryonic antigen (CEA) for destruction [52]. CEA is a cell surface glycoprotein that is overexpressed in the majority of carcinomas and has been implicated in tumour neoplasia [53]. Design of a ribozyme that specifically targeted CEA mRNA induced a 70%–90% reduction in transcript levels. Additionally, incorporation of the suicide herpes simplex thymidine kinase gene into the CEA-targeting ribozyme promoted mediated highly efficient and specific destruction of CEA expressing cancer cells following addition of the prodrug ganciclovir [52]. Whilst these studies have demonstrated a proof of principle for ribozyme-mediated *trans*-splicing in vitro, it has yet to be used successfully in vivo. Ribozymes are generally inefficient under physiological conditions because of their requirement for high Mg^2+^, although ribozyme modifications have improved their efficiency in mammalian cells [54]. Also, as these ribozymes are ∼400 nucleotides in length and the therapeutic 3′ exon sequences can be significantly larger, expression cassettes will be a necessity to deliver ribozymes in vivo, which in turn has all of the associated delivery issues.

## RNA Interference: Silencing of Specific mRNA Isoforms and Mutant Alleles

RNA interference (RNAi) is a highly conserved cellular mechanism of post-transcriptional gene silencing. Since its elucidation in 1998 [55], a more detailed understanding of its biochemical mechanism has revealed that the key effector molecules of RNAi are double-stranded small interfering RNAs (siRNAs) of approximately 21 nt in length [56]. These siRNAs can be artificially synthesised and delivered exogenously and also can arise endogenously via the transcription and processing of microRNA (miRNA) genes [57]. Depending upon their origin and the degree of sequence complementarity between siRNA and target mRNA, outcomes may vary between cleavage of the target mRNA and translational inhibition. In certain circumstances, siRNAs can direct the methylation of genomic DNA, thereby contributing to transcriptional silencing [58]. The intrinsic functions of RNAi, in particular those of miRNAs, are now known to be far-reaching, and to be fundamental for basic cellular processes during development, in differentiated tissues, and also in disease [59,60]. Exogenous activation of the RNAi pathway utilising synthetic siRNAs or siRNAs expressed from plasmid or viral vectors has become invaluable for gene knockdown in functional genomics and has significant therapeutic potential.

The power and promise of RNAi as a therapeutic modality lies in its intrinsic cellular mechanism and exquisite sequence specificity. In mechanistic terms, RNAi allows very different therapeutic options from conventional gene therapy. Aside from the knockdown of genes whose activity is implicated in disease pathogenesis, for example, in HIV [61], hepatitis B virus (HBV) [62], macular degeneration [63], Alzheimer disease [64], and cancer [65], the sequence specificity of RNAi allows the targeted knockdown of specific alternatively spliced isoforms and mutant disease alleles.

### 

#### 

##### Isoform-specific RNAi.

**Figure 3:**
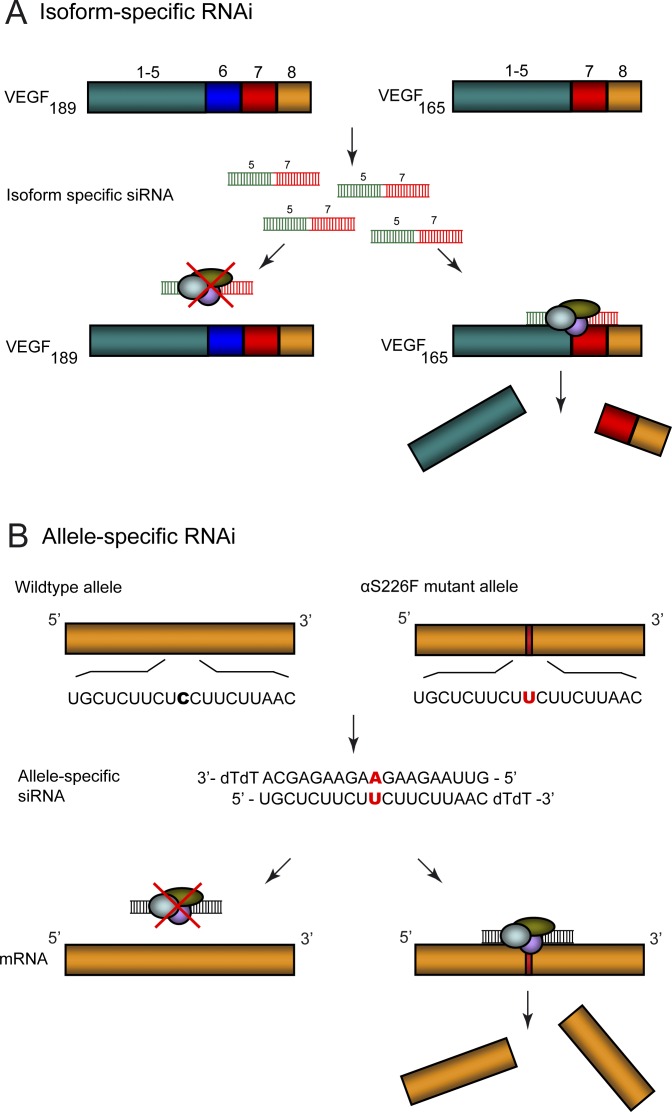
RNAi for Isoform- and Allele-Specific Silencing (A) Isoform-specific RNAi to target disease-associated isoforms in cancer. VEGF_165_ isoform overexpression is implicated in tumour angiogenesis. Targeting of the VEGF transcript with siRNA targeted to the exon 5/7 boundaries, in association with RISC, induces specific VEGF_165_ knockdown, while having no effect on other VEGF isoforms, e.g., VEGF_189_. (B) Allele-specific RNAi in the autosomal dominant slow channel congenital myasthenic syndrome. A missense mutation (red bar) in the muscle acetylcholine α-subunit (αS226F) induces a C→U change in the mutant allele. Use of siRNA specific for the αS226F mutation (A binding to U), induces discriminatory silencing of the mutant transcript, leaving the wild-type transcript mostly unaffected.

As many as 74% of all multi-exon human genes are thought to generate alternatively spliced transcripts [66]. This enhances proteome diversity; however, defects in this process are linked to numerous genetic diseases and cancer. Isoform-specific RNAi can be engineered with relative ease by targeting unique sequences within or at the boundaries of specific alternatively spliced exons (Figure 3A). This allows the specific knockdown of foetal, adult, tissue-specific, or disease-associated isoforms [67,68]; for example, the targeting of disease-associated isoforms in cancer. Five alternatively spliced VEGF isoforms exist, one of which, VEGF_165_, is strongly implicated in tumour angiogenesis. Silencing the VEGF_165_ isoform with RNAi by targeting unique sequences at the exons 5–7 boundary is not only feasible but leaves other functional VEGF isoforms intact [69]. Another example demonstrates the precision of RNAi to knockdown specific protein isoforms from among a large array of related isoforms with essential cellular functions. PI3-kinase, a critical signal transducer, comprises multiple isoforms and splice variants, one of which, the class Ia PI3-kinase catalytic alpha subunit, is implicated in tumour angiogenesis. Specific targeting of this isoform with RNAi has been demonstrated in a model of ovarian cancer [70].

In cases where aberrant splicing is associated with disease, isoform-specific knockdown by RNAi could be exploited to restore the balance between different isoforms or to target specific splicing regulators. Just as *trans*-splicing PTMs can be engineered to address the tau isoform imbalance in patients with frontotemporal dementia with parkinsonism linked to Chromosome 17, RNAi could be used to reprogram this balance by reducing the levels of exon 10+ splice variants. The recent identification of tau exon 10 splicing silencers, such as SRP54 [71], and enhancers [72,73] offers a different possibility for restoring normal tau splicing by directly targeting modulators of exon 10 splicing by RNAi. However, given that splicing is a tightly controlled process, down-regulation of endogenous splicing factors has the potential to significantly disrupt or alter splicing in nontargeted genes and off-target effects would need to be carefully monitored.

##### Allele-specific RNAi.

The exquisite specificity of RNAi also opens the possibility of targeting mutant alleles associated with dominant genetic disease, i.e., where the mutant allele is pathogenic in the presence of a normal allele [74–76] (see Figure 3B). This approach permits discrimination between wild-type and mutant alleles, leaving the former largely intact, an important consideration given that in many cases these are essential genes. Critical to this is the capacity of the RNAi machinery to discriminate single nucleotide mismatches among closely related gene targets [77]. Original approaches placed mismatches around central positions 10 and 11 in the siRNA duplex corresponding to nucleotides in the target mRNA cleaved by the EIF2C2 (previously known as Ago2) component of the RNA-induced silencing complex (RISC)[78,79]. It is now appreciated that both the location and type of nucleotide mismatch influence allele-specific discrimination. Du et al. reported significant tolerance for mismatches at positions 1–4 and 12–19, i.e., mismatches at these positions offer poor discrimination [80]. Schwarz et al. confirmed some of these findings, reporting that mismatches in the 5′ “seed” region (i.e., positions 2–7) are weakly selective but that mismatches at other positions, notably centrally and also at position 16, are powerfully discriminative [81]. Thus, there is a need for further studies to establish to what extent general rules exist, and also to extend these findings to expressed siRNA sequences.

Allele-specific silencing of several mutant allele targets has been studied for diseases including osteogenesis imperfecta [82], sickle cell anaemia [83], primary retinal degeneration [84], and spinocerebellar ataxia [85]. By designing both siRNAs and shRNAs with the AChR αS226F mismatch mutation placed at position 10 in the duplex, significant discrimination between mutant and wild-type knockdown was demonstrated, as determined at the protein level by sensitive radio-labelling and other measures [86]. Studies by several groups have targeted the G85R and G93A mutations in the Cu/Zn superoxide dismutase *(SOD1)* gene. Mismatches either located centrally at position 10 [87], or more 3′ [88], provide good discrimination in vitro. The feasibility of extending this in vivo has been demonstrated by silencing a human *SOD1* G93A transgene in mice using lentiviral expression, although these experiments did not show allele-specific silencing [89,90]. In another example, a GAG deletion in the *TOR1A* gene causes dominantly inherited dystonia. Placement of a trinucleotide GAG mutation at or near the centre of the siRNA duplex induces selective silencing of the mutant gene in vitro in Cos-7 cells expressing both wild-type and mutant *TOR1A* [91]. Thus, point mutations and small deletions appear amenable to an allele-specific strategy. For larger mutations, including those of the polyglutamine diseases, where extended CAG repeats are the common feature, an alternative approach targeting a SNP linked to the mutation seems promising. Targeting of a G→C SNP immediately 3′ to the CAG repeat in the *MJD1* disease allele that causes spinocerebellar ataxia type 3, shows effective discrimination between silencing of the wild-type and mutant alleles [92]. The effectiveness of this approach ultimately depends on tight linkage between the target SNP and disease allele; in this particular case, over 70% of disease alleles are linked to the C variant [92]. There is strong evidence to indicate that silencing the mutant allele in the most common polyglutamine disease, Huntington disease, is likely to be feasible in vivo and of therapeutic value [93–95]. However, as yet, there are no reports of allele-specific discrimination between mutant and wild-type huntingtin. Continued optimisation of targeting constructs, mismatch positions, and further identification of disease linked SNPs, especially in the case of Huntington disease, will facilitate progress in this area.

##### RNAi limitations.

While RNAi offers novel therapeutic applications and can obviate some of the short-comings of conventional gene therapy, it is not without its own limitations. Delivery is a major obstacle to the clinical exploitation of RNAi therapies. The delivery of synthetic siRNAs requires improvement via chemical stabilisation and the development of targeting methods, but in many respects these molecules can be treated and optimised as conventional pharmaceutical agents. In contrast, virally expressed siRNAs retain the advantages but also many of the limitations of standard gene therapy vector delivery systems. In recent years, a growing understanding of RNAi biology has allowed improved siRNA sequence selection and also the incorporation of nucleotide modifications to facilitate guide-strand loading into RISC [96,97]. Nevertheless, nonspecific off-target effects, whether due to limited sequence homologies or to miRNA-like effects, continue to be of significant concern [98]. In the case of siRNAs, it is now thought that specific chemical modifications might abolish or limit these effects [99,100]. For expressed siRNAs, vector capacity is not the limiting factor that it can be for conventional gene therapy; however, the regulatory elements in these vectors require improvement. Most studies currently utilise Pol III promoters, commonly U6 or H1, to drive siRNA expression, but these lack the possibilities for spatial or temporal regulation. Increasingly, the use of miRNA-like siRNAs will permit the evaluation of Pol II promoters to drive tissue-specific regulation [101]. Finally, while expressed siRNAs are likely to elicit similar immunological responses directed against vector components to those reported in standard gene therapy studies, synthetic siRNAs have their own set of inflammatory concerns. A number of studies have reported siRNA-induced interferon responses, originally thought to be associated with longer dsRNAs [102–104]. It is now known that specific nucleotide motifs in dsRNAs can activate the immune system via Toll-like receptors (TLRs), specifically TLR3, TLR7, TLR8, and TLR9 [105,106]. Where characterised, such motifs can be avoided in siRNA design but it is also now known that chemical modification at specific nucleotides can abolish TLR recognition and enhance safety [107].

**Box 1. Obstacles to Successful Clinical Application of Genetic Therapies**

**Conventional Gene Therapy**

- Expression of transgene is not regulated by endogenous factors
- Gene replacement does not address alternative splicing and multiple isoforms
- Size of transgene limited by delivery vectors
- Possibility of insertional mutagenesis with some vectors
- Autosomal dominant disorders not amenable to gene replacement
- Immune response to viral vector proteins limits readministration of the transgene

**RNA-Based Therapy**

- Systemic delivery of nucleic acid may require carrier system to improve transfection efficacy and protect from nuclease degradation
- Non-permanent correction of mRNA will necessitate readministration
- Binding to nonspecific targets may elicit unwanted side effects
- AO/siRNA competitive binding to endogenous splicing machinery or miRNA pathway components
- Possible induction of interferon immune response to RNAi

## Delivery

The major obstacle to the successful application of all RNA-based therapies is delivery to target tissues, a problem further complicated by the potential for rapid degradation by cellular nucleases. Chemically stabilised forms of antisense oligonucleotides, e.g., morpholino oligomers and peptide nucleic acids, and siRNAs, have been developed in efforts to prolong half-lives and enhance bioavailability. However, for most applications, re-administration will remain a necessity. Given the challenges that face nucleic acid delivery, it is likely that applications involving local rather than systemic delivery will be the first to be evaluated for success in clinical application. For the majority of nucleic acid chemistries, complexation or covalent linkage to specialised delivery agents will be necessary to facilitate cellular uptake. Lipid-based delivery agents have been combined with chemically modified siRNAs to successfully target HBV in a mouse model of HBV replication [62], and further advances in the lipid-based agents are being developed by many groups [108]. The use of nucleic acids directly conjugated to targeting ligands is another promising approach to enhance delivery. For example, conjugation of cholesterol to the 3′ end of the sense strand of siRNAs targeting apolipoprotein allows efficient liver delivery and effective silencing in vivo [109]. More recently, lipid targeting complexes incorporating apolipoprotein A-I targeting ligands have proved highly effective for single, low dose siRNA delivery to liver and effective gene silencing in an HBV model [110]. Advances in nanoparticle technology should eventually lead to intelligent delivery systems that contain stabilised nucleic acid cargoes within polymer complexes incorporating specific targeting ligands [111]. Finally, in some cases, the use of viral expression systems will be possible and appropriate depending on the application. For example, the expression of U7 small nuclear RNA–based constructs using AAV vectors has proved highly successful for exon skipping in a mouse model of DMD [36], and a number of siRNA expression systems using short hairpin RNA- or microRNA-based constructs have been successfully delivered using AAV [85], lentivirus [112], and other vector systems. However, viral delivery of RNA therapeutics will share the same challenges of more conventional gene replacement therapies, including immune response to viral vectors.

## Conclusion

RNA directed therapy offers a range of specialised applications not available to conventional gene therapy. However, promising as such applications appear, their efficacy has yet to be convincingly demonstrated in clinical trials, and their success is currently limited by a number of factors (Box 1). In the absence of expression systems, oligonucleotide therapies are unlikely to offer permanent correction and will therefore require re-administration. The use of more stable nucleic acid chemistries should allow a reduction in the frequency of dosing, but the long-term consequences of such re-administration protocols are not yet known. Another significant limitation of most RNA targeting applications involving oligonucleotides are nonspecific off-target effects. While the lengths of such nucleic acids are generally sufficient to confer target recognition specificity, the incorporation or targeting of common nucleotide motifs (e.g., TLR recognition sites, miRNA binding sites, and ESEs) means that there is a potential for nonspecific events. Having said this, and despite such limitations, progress in the field of RNA therapeutics over the last decade has been remarkable. A large number of antisense oligonucleotide agents are in clinical development, including those for specialised applications such as exon skipping and splicing modification. Phase I and II clinical trials for exon skipping in DMD are underway in The Netherlands and will start shortly in the United Kingdom. Therapeutic RNAi developments are not far behind, with local delivery applications, e.g., Sirna-027 treatment of neovascularization associated with age-related macular degeneration (http://www.clinicaltrials.gov), the first to be underway. The realisation of the potential of RNA targeted therapies to address genetic disease suggests that this field has a very promising future.

## Abbreviations

AAV: adeno-associated virus
AO: antisense oligonucleotide
CEA: carcinoembryonic antigen
CF: cystic fibrosis
*CFTR*: cystic fibrosis transmembrane conductance regulator
DMD: Duchenne muscular dystrophy
*DMPK*: dystrophia myotonica-protein kinase
ESE: exon splicing enhancer
HBV: hepatitis B virus
**MAPT**: microtubule-associatedprotein tau
miRNA: microRNA
PTM: pre-mRNA *trans*-splicing molecule
RISC: RNA-induced silencing complex
RNAi: RNA interference
siRNA: small interfering RNA
SMA: spinal muscular atrophy
SMaRT: spliceosome mediated RNA *trans*-splicing
*SMN*: survival motor neuron
TLR: Toll-like receptor
